# Lateral dislocation of the knee joint after total knee arthroplasty: a case report

**DOI:** 10.1186/1757-1626-1-75

**Published:** 2008-08-08

**Authors:** Ender Ugutmen, Korhan Ozkan, Koray Unay, Mahir Mahirogullari, Engin Eceviz, Omer Taser

**Affiliations:** 1 Department of Orthopaedics and Traumatology, Goztepe Training and Research Hospital, Istanbul, Turkey; 2 Department of Orthopaedics and Traumatology, Gülhane Military Medical Academy Faculty of Medicine, Istanbul, Turkey; 3 Department of Orthopaedics and Traumatology, Istanbul University Istanbul Faculty of Medicine, Istanbul, Turkey

## Abstract

**Background:**

Total knee arthroplasty (TKA) is a successful therapy for functional improvement and pain relief in advanced symptomatic degeneration of the knee joint. But it can be associated with many complications, one of which is instability.

**Case presentation:**

A 70-year-old woman was referred to our hospital because of right knee dislocation after TKA was performed on her right knee due to severe varus deformity and flexion contracture. This instability was caused by persistent MCL tightness and iatrogenic lateral collateral, arcuate ligament, and popliteus tendon injury.

The torn lateral collateral ligament and arcuate ligament were sutured with no. 2 non-absorbable (Ethibond) sutures with plication of the posterolateral knee capsule. A deep-dish liner was inserted to optimize soft tissue tension.

**Conclusion:**

This is a very severe complication, and surgeons must be cautious about ligament balancing and soft tissue resection during TKA for severe varus and valgus deformities.

## Background

Total knee arthroplasty (TKA) is a successful therapy for functional improvement and pain relief in advanced symptomatic degeneration of the knee joint [1]. But it can be associated with many complications, one of which is instability [2].

We describe a case of postoperative lateral knee dislocation after TKA. To our knowledge, there have been case reports in the English literature about anterior and posterior knee dislocation after TKA but not about lateral dislocation [3].

In this report, we aimed to investigate the reason for this type of complication, describe the required treatment modality, and emphasize the importance of ligamentous balancing during TKA.

## Case presentation

**Figure 1:**
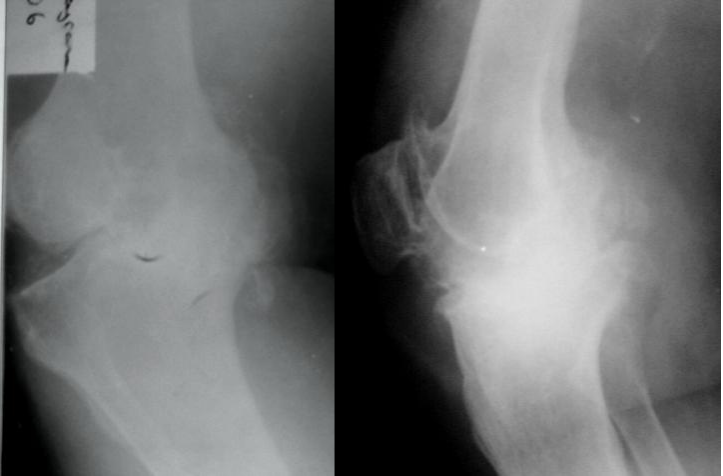
**AP and lateral roentgenography of the knee before the first operation**.

**Figure 2:**
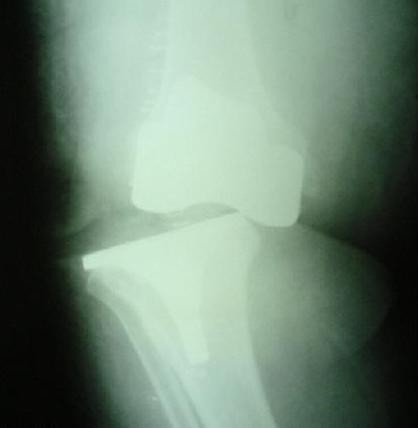
AP roentgenography of the knee at postoperative second day.

A 70-year-old woman was referred to our hospital because of right knee dislocation after TKA was performed on her right knee due to severe varus deformity and flexion contracture (figure 1). Before TKA, the patient had a 45° flexion contracture and severe varus deformity, according to the patient's records. The patient's knee had dislocated when she first tried to stand and walk on the second day after surgery. The day after the dislocation, the patient was referred to our institute (figure 2). We attempted closed reduction of the dislocated knee in the operating room, but this was unsuccessful. In the first operation, size 5 femoral and size 4 tibial components (Genesis II, Smith & Nephew) of a non-posterior-stabilized prosthesis with a size 11 liner were inserted. Paramedian arthrotomy was performed via the previous longitudinal incision, and the knee was exposed. Eversion and lateral dislocation of the patella were attempted; however, the surrounding soft tissues were very tight and further proximal dissection and patellofemoral ligament resection was required to enable patellar eversion and knee flexion to 90°. The liner was then removed. After placing the retractors in the appropriate sites, we observed that the lateral collateral ligament, arcuate ligament, and popliteus tendon were severely torn, with inadequate release of the medial structures, including the medial collateral ligament (MCL) and intact medial sleeve with pes anserine tendons. The tibia dislocated laterally, even in the absence of a laterally applied force. First, a periosteal elevator was used to strip the superficial MCL from the proximal tibia, up to the diaphyseal region. Subsequently, the insertion of the pes anserine tendon was released with a cautery. The posterior capsule was released from the top of the tibia and the back of the posteromedial femur with an electrocautery and the curved tip of the osteotome, respectively. A second incision was made, extending from the lateral femoral condyle to the head of the fibula. Since grade III acute injury of the posterolateral corner of the knee was present, we preferred direct repair of the injured structures [4]. Upon incision of the fascia between the iliotibial tract and the biceps femoris, midsubstance tears of the lateral collateral ligament, arcuate ligament, and popliteus tendon were revealed. These were treated by performing an end-to-end repair with Bunnell-type crossing sutures [5]. The popliteus tendon was also found to be avulsed from the femur, and transosseous sutures were used to attach the tendon to its proximal insertion site. Posterolateral capsular repair and plication were performed, and the tendon of the lateral gastrocnemius muscle was sutured to the adjacent capsule. Subsequently, a size 15 deep-dish liner was inserted in order to optimize soft tissue tension. Rotational alignments of the tibial and femoral components were normal so they were not revised; further, there was no malalignment of the limb after the reduction of the knee prosthesis.

**Figure 3:**
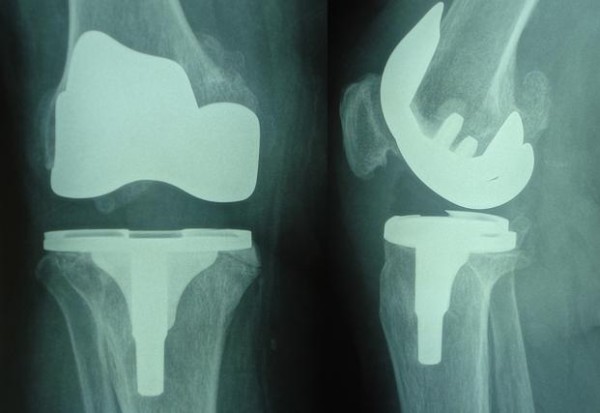
AP and lateral roentgenography of the knee at postoperative 14 months.

During knee flexion, the patella subluxated laterally; hence, lateral retinacular release was performed. Furthermore, 2 suction drains were applied for 4 days until hematoma drainage ceased, and the patient's knee was kept in a brace for 2 months. Culture of the synovial fluid that was collected during the second operation revealed methicillin-resistant *Staphylococcus aureus *(MRSA); therefore, parenteral teicoplanin (Targocid^®^) treatment was started. Antibiotic therapy was continued for 1 month, and the eradication of the infection was confirmed clinically and with the help of laboratory tests. During this period, the patient was enrolled in a physical therapy program that included. Only isometric quadriceps exercises with no flexion and walking without weight bearing for 1 month. After 1 month, range of movement (ROM) exercises was begun, and the patient started to walk with full weight bearing and was able to achieve full knee extension. After the end of 14 postoperative months, the patient had a knee flexion of 100° and no varus or valgus instability (Figure 3).

## Discussion

This was a case of asymmetric extension instability due to preoperative severe varus angular deformity of the knee; this instability was caused by persistent MCL tightness and iatrogenic lateral collateral, arcuate ligament, and popliteus tendon injury [2]. Although anterior and posterior dislocation after TKA has been reported, we have not found any case reports regarding acute lateral dislocation after TKA in the English literature; we did however find some report of post-TKA lateral subluxation [3,7]. The dislocated knee was first tried to be reduced under general anesthesia in order to prevent vascular or neurological insult. Reducing the knee would also give us the opportunity to make a surgery in a normally aligned knee which would probably decrease the possibility of the iatrogenic nerve or vascular injury.

In our case, instead of using a constrained implant, keeping in mind that asymmetric extension instability occurs due to insufficient medial release after TKA in a knee with preoperative varus deformity, we attempted to achieve satisfactory soft tissue balance with adequate medial release and by using a thicker tibial insert. Further, since iatrogenic acute grade III injury of the posterolateral corner was present, we preferred direct anatomic repair of the injured structures [4]. Nevertheless, the usual technique for dislocation of knee joint after knee arthroplasty is a constrained implant and isolated ligament reconstructions and polyethylene insert exchanges can fail to restore stability to the knee [7]. Also, constrained prosthesis would have allowed early range of motion and immediate full weight bearing in our patient.

One disastrous complication of a recurrent dislocation of total knee arthroplasty is also a vascular complication even can result in an above-knee amputation [8], so surgeons must be extremely cautious about ligament balancing and soft tissue resection during TKA for severe varus and valgus deformities [7,9,10].

## Conclusion

This complication occured due to the inadequate release of tight MCL with iatrogenic lateral collateral, arcuate ligament, and popliteus tendon injury during total knee arthroplasty to a knee with severe varus angular deformity. 14 months postoperatively, our patient is well with independent walking but longer follow up of is necessary to completely assess the success of our treatment.

## Abbreviations

TKA:Total knee arthroplasty; MCL: Medial collateral ligament; MRSA: methicillin-resistant *Staphylococcus aureus*; ROM: Range of motion.

## Competing interests

The authors declare that they have no competing interests.

## Authors' contributions

KO and EU wrote the case report including performing the literature review. KU and EE were involved in the literature review and helped draft part of the manuscript. MM and OT critically reviewed the manuscript. supervised the writing and the general management of the patient. All authors read and approved the final manuscript.

## Consent

Written informed consent was obtained from the patient for publication of this case report and accompanying images. A copy of the written consent is available for review by the Editor-in-Chief of this journal.
